# Dietary Fat Patterns and Outcomes in Acute Pancreatitis in Spain

**DOI:** 10.3389/fmed.2020.00126

**Published:** 2020-04-09

**Authors:** Guillermo García-Rayado, Gregorio Varela-Moreiras, Ángel Lanas, Ángel Ferrández, Nelly Balza-Lareu, Juan I. Cervera, María P. Bodenlle-Bello, Ana M. Argüelles-Arias, Patricia Latorre, María A. Udaondo-Cascante, María J. Soria-de-la-Cruz, José Lariño-Noia, Roberto García-Figueiras, Cristina Gil-García-Ollauri, Ricardo Ituarte-Uriarte, Carmen L. Rosales-Alexander, Jordi Soriano, María Rodríguez-Peláez, Alicia Mesa-Álvarez, Elida Oblitas, María M. Menso, Federico Bertoletti, José I. Rodríguez-Prada, Silvia Guzmán-Suárez, Daniel Closa, Enrique de-Madaria

**Affiliations:** ^1^Service of Digestive Diseases, University Clinic Hospital Lozano Blesa, Aragón Health Research Institute (IIS Aragón), CIBERehd, Zaragoza, Spain; ^2^Department of Pharmaceutical & Health Sciences, Universidad CEU San Pablo, Madrid, Spain; ^3^Department of Gastroenterology, Hospital Son LLatzer, Palma, Spain; ^4^Department of Radiology, Hospital Clínico Universitario, Valencia, Spain; ^5^Department of Radiology, Hospital Meixoeiro, Vigo, Spain; ^6^Department of Radiology, Hospital Universitario Vírgen Macarena, Seville, Spain; ^7^Department of Gastroenterology, Hospital Universitario Doctor Peset, Valencia, Spain; ^8^Department of Radiology, Hospital Clínico Universitario, Valladolid, Spain; ^9^Department of Gastroenterology, Hospital Puerta del Mar, Cádiz, Spain; ^10^Department of Gastroenterology and Hepatology, University Hospital of Santiago de Compostela, Santiago de Compostela, Spain; ^11^Department of Radiology, University Hospital of Santiago de Compostela, Santiago de Compostela, Spain; ^12^Department of Gastroenterology, Hospital Universitario Cruces, Barakaldo, Spain; ^13^Department of Radiology, Hospital Universitario Cruces, Barakaldo, Spain; ^14^Department of Gastroenterology, Hospital Universitari Doctor Josep Trueta, Girona, Spain; ^15^Department of Gastroenterology, Hospital Universitario Central de Asturias, Oviedo, Spain; ^16^Department of Radiology, Hospital Universitario Central de Asturias, Oviedo, Spain; ^17^Department of Gastroenterology, Hospital de la Santa Creu i Sant Pau, Barcelona, Spain; ^18^Department of Radiology, Hospital de la Santa Creu i Sant Pau, Barcelona, Spain; ^19^Department of Gastroenterology, Hospital Xeral, Vigo, Spain; ^20^Department of Surgery, Hospital Xeral, Vigo, Spain; ^21^Department of Experimental Pathology, Institut d'Investigacions Biomèdiques de Barcelona (IIBB-CSIC-IDIBAPS), Barcelona, Spain; ^22^Department of Gastroenterology, Alicante University General Hospital, Alicante Institute for Health and Biomedical Research (ISABIAL-FISABIO Foundation), Alicante, Spain

**Keywords:** acute pancreatitis, diet, fat intake, obesity, unsaturated fatty acids

## Abstract

**Background/Objective:** Evidence from basic and clinical studies suggests that unsaturated fatty acids (UFAs) might be relevant mediators of the development of complications in acute pancreatitis (AP). Objective: The aim of this study was to analyze outcomes in patients with AP from regions in Spain with different patterns of dietary fat intake.

**Materials and Methods:** A retrospective analysis was performed with data from 1,655 patients with AP from a Spanish prospective cohort study and regional nutritional data from a Spanish cross-sectional study. Nutritional data considered in the study concern the total lipid consumption, detailing total saturated fatty acids, UFAs and monounsaturated fatty acids (MUFAs) consumption derived from regional data and not from the patient prospective cohort. Two multivariable analysis models were used: (1) a model with the Charlson comorbidity index, sex, alcoholic etiology, and recurrent AP; (2) a model that included these variables plus obesity.

**Results:** In multivariable analysis, patients from regions with high UFA intake had a significantly increased frequency of local complications, persistent organ failure (POF), mortality, and moderate-to-severe disease in the model without obesity and a higher frequency of POF in the model with obesity. Patients from regions with high MUFA intake had significantly more local complications and moderate-to-severe disease; this significance remained for moderate-to-severe disease when obesity was added to the model.

**Conclusions:** Differences in dietary fat patterns could be associated with different outcomes in AP, and dietary fat patterns may be a pre-morbid factor that determines the severity of AP. UFAs, and particulary MUFAs, may influence the pathogenesis of the severity of AP.

## Introduction

Acute pancreatitis (AP) is a common health issue (1). While most patients have a mild disease course, one-third experience local and/or systemic complications that produce an increase of morbidity (2). Furthermore, patients with systemic complications have a higher mortality, which is as high as 50% among patients with persistent (>48 h) organ failure (POF) (2). The factors that determine the development of complications in AP are currently poorly understood. Obesity, particularly visceral adiposity, is associated with local complications, with systemic complications, and with increased risk of death (3–5). *In vitro* experiments using acinar cells of the pancreas have shown that unsaturated fatty acids (UFAs) are associated with inflammation and necrosis, while saturated fatty acids (SFAs) are not harmful. These experiments suggest that UFAs produce necrosis of acinar pancreatic cells and uncontrolled UFA release results in high UFA levels in the bloodstream and is associated with kidney failure and with cell damage in lung alveoli (6, 7). UFAs have been detected in pancreatic necrosis collections (6–9). Furthermore, oleic acid chlorohydrin, a halogenated product derived from monounsaturated oleic acid, was recently described as a blood marker and mediator of complications in a rat model of AP (10) and in patients with AP (11). Thus, UFA-mediated toxicity seems to be a key pathway in determining complications of AP (11, 12). Preceding studies have suggested that diet affects body fatty acid composition. In animal models, mice with diets with different content in oleic acid and linoleic acid show these differences on the composition of fatty acids of their tissues. Studies in humans also show this association (13, 14). Consequently, it is possible that the changes induced by dietary UFAs can modulate the severity of AP. To our knowledge, there are no clinical studies studying this relationship. This study aimed to compare clinical course of disease of AP in regions of Spain that have different dietary fat intake patterns.

## Materials and Methods

This was a retrospective analysis of data from a prospective cohort study, the Atlantis Project, plus data from a cross-sectional study, the ANIBES (Anthropometric data, macronutrients and micronutrients intake, practice of physical activity, socioeconomic data, and lifestyles in Spain) study.

The Atlantis Project was endorsed by the Spanish Association of Pancreatology (AESPANC) and by the Spanish Association of Gastroenterology (AEG). It aimed to validate and compare the determinants of AP severity and the severity classifications of AP (2) as well as to ascertain the role of comorbidity in the course of AP (4). Described in detail elsewhere (2), the Atlantis project was a 23-center nationwide prospective study which analyzed 1,655 patients with AP recruited from June 2013 to February 2015. All centers were tertiary care hospitals with availability of critical care. In Spain, there is a public healthcare system and citizens have similar access with similar quality of care in the different regions of the country. The project was carried out following the rules of the Declaration of Helsinki of 1975. The study required written informed consent and was approved by the Institutional Review Boards (IRBs) of the participating centers. This new analysis was also approved by the central IRB of the Atlantis study, namely the *Comité Ético de Investigación con Medicamentos del Hospital General Universitario de Alicante (PI2018-102)*. For the diagnosis of AP, two or more criteria had to be fulfilled: (A) typical upper abdominal pain; (B) serum amylase and/or lipase levels that were at least three times greater than the upper limit of normal; and (C) imaging compatible with AP (15). Patients with a diagnosis of chronic pancreatitis were excluded. Patient outcomes were recorded during their hospital stay, and the following data were retrieved from the study database for this analysis: local complications (acute peripancreatic fluid collections or pancreatic and/or peripancreatic necrosis), POF, mortality (in-hospital mortality), and disease severity (moderate-to-severe disease). These were defined according to the revised Atlanta classification (15). Obesity was defined as body mass index ≥30 kg/m^2^.

The aim of the ANIBES study was to update the food and beverage intake, behavior, and anthropometric measurements of the Spanish citizens, as well as to determine their energy expenditure and physical exercise habits (sample: 2009 citizens) (16). Diet was studied using a dietary diary (during 3 days) recorded on a tablet device plus a 24-h dietary recall. The participants were explained that the diet during those 4 days had to be representative of their diet of the past years. The ANIBES study divided Spain into nine regions: Barcelona, Canary Islands, Central, Levant, Madrid, North-East, North-West, North-Central, and South (16). Data regarding the consumption of lipids, SFAs, monounsaturated fatty acids (MUFAs), and polyunsaturated fatty acids (PUFAs) were retrieved from the original ANIBES database. According to ANIBES, the Spanish mean daily caloric intake is 1,810 kcal/day, including 38.5% from lipids (overall), 11.7% from SFAs, and 23.43% from UFAs (16.8% from MUFA and 6.63% from PUFAs) (17). Patients in the Atlantis Project from regions that had intake levels that were higher than the national mean values were considered to be high consumers regarding that specific type of fatty acid, while patients from regions that had intake levels that were lower than the Spanish mean values were classified as low consumers. For example, the national mean intake of UFAs is 23.6% (mean percentages of total caloric intake) and the mean intake of UFAs in North-Central region of Spain is 24.2%. Therefore, patients in the Atlantis Project from North-Central region were considered to be high consumers of UFAs. The differences in consumption of PUFAs in the different regions of Spain are minimal, so we have not included this type of fatty acid in the analysis.

### Statistics

Continuous data were analyzed for normality by the Shapiro-Wilk test and were summed-up using means and standard deviations or medians and interquartile ranges (IQRs) depending on the variable distribution. Qualitative variables were expressed as number (*n*) and percentage (%). The association of low or high fat consumption according to region and outcome was investigated using a chi-square test or Fisher's exact test if necessary and using binary logistic regression analysis for multivariable analysis. Two multivariable analysis models were used: (1) a model without obesity that included age, comorbidity (according to the Charlson comorbidity index, cutoff ≥3) (18), sex, alcoholic etiology, and recurrent AP (≥1 previous episode); (2) a model with obesity that included all of these variables plus obesity. The adjusted odds ratios (ORs) were calculated. Statistical calculations were done using SPSS 21.0 (IBM, Armonk, NY, USA).

## Results

Table 1 shows the detailed baseline characteristics and outcomes of the 1,655 patients with AP. The patients had a median age of 66 years; 54% were male, 24% were obese, 60% had gallstone etiology, 27% had local complications, 7% had POF, and 4% died. Regarding these baseline characteristics, there were no significant differences between the different regions of Spain (*p* > 0.05). Supplementary Table 1 shows the caloric profile of lipid intake of the Spanish population as stratified by region according to the ANIBES study. Supplementary Table 2 lists the number of medical centers and patients of the Atlantis database in the regions defined in the ANIBES study.

**Table 1:** Baseline characteristics and outcomes of patients with acute pancreatitis.

**Characteristics and outcomes**	**Overall**
*N*	1,655
Age, years; median (IQR)	66 (51–79)
Male sex, *n* (%)	891 (53.8%)
**BMI, kg/m**^**2**^	
Median (IQR)	26.8 (24.3–29.7)
BMI < 25	509 (31.5%)
BMI 25– <30 (Pre-Obesity)	722 (44.7%)
BMI ≥ 30 (Obesity)	383 (23.7%)
CCI, points; median (IQR)	3 (1–5)
Liver disease, *n* (%)	120 (7.2%)
**Etiology**, ***n*** **(%)**	
Gallstones	984 (59.5%)
Alcohol	251 (15.2%)
Idiopathic	235 (14.2%)
Other	185 (11.2%)
Recurrent AP, *n* (%)	422 (25.5%)
**Local complications**, ***n*** **(%)**	
Any local complication	444 (26.8%)
APFC	163 (9.8%)
Peri(pancreatic) necrosis	281 (17%)
Persistent organ failure, *n* (%)	113 (6.8%)
Mortality, *n* (%)	70 (4.2%)

*IQR, interquartile range (Q1–Q3); BMI, body mass index; CCI, Charlson comorbidity index; AP, acute pancreatitis; APFC, acute peripancreatic fluid collection; Peri(pancreatic) necrosis, pancreatic and/or peripancreatic necrosis*.

### Univariate Analysis

Table 2 shows outcomes according to lipid intake (univariate analysis). Moderate-to-severe disease was more frequently present in patients from regions with a high overall lipid intake [37 vs. 32.1%, *p* = 0.04], who also showed a non-significant trend toward a higher frequency of local complications [28.4 vs. 24.6%, *p* = 0.08]. Patients from regions with high intake of UFAs had significantly more local complications [28.6 vs. 20.5%, *p* = 0.002], POF [7.7 vs. 3.8%, *p* = 0.01], mortality [4.8 vs. 2.2%, *p* = 0.03], and moderate-to-severe disease [36.7 vs. 29%, *p* = 0.007]. Being from a region with high MUFA intake was significantly associated with more local complications [29.6 vs. 23.9%, *p* = 0.009] and moderate-to-severe disease [38.1 vs. 31.7%, *p* = 0.007]. SFA intake had no effect on outcomes (Table 2).

**Table 2:** Outcomes of acute pancreatitis according to regional lipid intake.

**Lipid**	**Intake**	**Outcomes:** ***n*** **(%)**			
		**Local complications**	**Persistent organ failure**	**Mortality**	**Moderate-to-severe AP**
Lipids (overall)	High	278 (28.4%)	71 (7.3%)	43 (4.4%)	362 (37%)
	Low	166 (24.6%)	42 (6.2%)	27 (4%)	217 (32.1%)
	*P*	0.08	0.4	0.7	**0.04**
SFAs	High	327 (26.9%)	79 (6.5%)	47 (3.9%)	430 (35.4%)
	Low	117 (26.7%)	34 (7.7%)	23 (5.2%)	149 (33.9%)
	*P*	0.9	0.4	0.2	0.6
UFAs	High	369 (28.6%)	99 (7.7%)	62 (4.8%)	473 (36.7%)
	Low	75 (20.5%)	14 (3.8%)	8 (2.2%)	106 (29%)
	*P*	**0.002**	**0.01**	**0.03**	**0.007**
MUFAs	High	252 (29.6%)	65 (7.6%)	39 (4.6%)	324 (38.1%)
	Low	192 (23.9%)	48 (6%)	31 (3.9%)	255 (31.7%)
	*P*	**0.009**	0.2	0.5	**0.007**

*Percentages are the proportions of the patients of the whole cohort (high or low consumers) that show each outcome of AP*.

*SFAs, saturated fatty acids; UFAs, unsaturated fatty acids; MUFAs, monounsaturated fatty acids; High, patients from a region of Spain with a proportion of caloric intake from all lipids or from the indicated types of lipids that was greater than the Spanish national mean; Low, patients from a region of Spain with a proportion of caloric intake from all lipids or from the indicated types of lipids that was lower than the Spanish national mean; AP, acute pancreatitis; P, P-value in the chi-square test (Fisher's exact test was not required)*.

*Bold value indicates P-value statistically significant*.

### Multivariable Analysis

In multivariable analysis (Table 3), patients from regions with high overall lipid intake had significantly more frequent moderate-to-severe disease in the model without obesity [OR = 1.26; 95% CI: 1.02–1.55; *p* = 0.034], but high overall lipid intake was not significant after obesity was included in the model [OR = 1.19; 95% CI: 0.96–1.48; *p* = 0.1]. Patients with AP from regions with high UFA intake had significantly higher rates of local complications [OR = 1.53; 95% CI: 1.15–2.05; *p* = 0.004], POF [OR = 2.1; 95% CI: 1.18–3.74; *p* = 0.01], mortality [OR = 2.37; 95% CI: 1.12–5.03; *p* = 0.02], and moderate-to-severe disease [OR = 1.42; 95% CI: 1.1–1.85; *p* = 0.007] in the model without obesity compared to patients from areas with low UFA intake; after entering the variable obesity into the model, high UFA intake remained a risk factor for POF [OR = 2.4; 95% CI: 1.24–4.71; *p* = 0.01], showing a non-significant trend for the other outcomes. Patients from regions with high MUFA intake had significantly increased rates of local complications [OR = 1.3; 95% CI: 1.03–1.62; *p* = 0.025] and moderate-to-severe disease [OR = 1.33; 95% CI: 1.08–1.63; *p* = 0.008], and the significance remained for moderate-to-severe disease after entering the variable obesity into the model [OR = 1.26; 95% CI: 1.02–1.56; *p* = 0.035]. SFA intake was not associated with any effect on outcomes (Table 3).

**Table 3:** Multivariate analysis of high overall lipid intake and lipid subtype intake and outcomes in acute pancreatitis.

**Lipid**	**Model**	**Adjusted odds ratio**			
		**Local complications**	**Persistent organ failure**	**Mortality**	**Moderate-to-severe AP**
Lipids (overall)	Without obesity	1.21 (0.96–1.53) *P =* 0.1	1.21 (0.82–1.8) *P =* 0.3	1.15 (0.7–1.88) *P =* 0.6	1.26 (1.02–1.55) ***P=*** **0.034**
	With obesity	1.11 (0.88–1.41) *P =* 0.4	1.23 (0.81–1.87) *P =* 0.3	1.11 (0.66–1.87) *P =* 0.7	1.19 (0.96–1.48) *P =* 0.1
SFAs	Without obesity	0.98 (0.76–1.26) *P =* 0.9	0.87 (0.57–1.32) *P =* 0.5	0.76 (0.45–1.28) *P =* 0.3	1.06 (0.84–1.3) *P =* 0.63
	With obesity	1.01 (0.78–1.3) *P =* 0.9	0.87 (0.57–1.34) *P =* 0.5	0.83 (0.48–1.42) *P =* 0.49	1.11 (0.88–1.4) *P =* 0.4
UFAs	Without obesity	1.53 (1.15–2.05) ***P=*** **0.004**	2.1 (1.18–3.74) ***P=*** **0.01**	2.37 (1.12–5.03) ***P=*** **0.02**	1.42 (1.1–1.85) ***P=*** **0.007**
	With obesity	1.34 (0.99–1.83) *P =* 0.06	2.4 (1.24–4.71) ***P=*** **0.01**	2.09 (0.94–4.66) *P =* 0.07	1.27 (0.96–1.67) *P =* 0.09
MUFAs	Without obesity	1.3 (1.03–1.62) ***P=*** **0.025**	1.36 (0.92–2) *P =* 0.1	1.29 (0.79–2.09) *P =* 0.3	1.33 (1.08–1.63) ***P=*** **0.008**
	With obesity	1.2 (0.95–1.5) *P =* 0.1	1.37 (0.91–2.06) *P =* 0.1	1.24 (0.75–2.07) *P =* 0.4	1.26 (1.02–1.56) ***P=*** **0.035**

*In the binary logistic regression analysis, the data are reported as adjusted odds ratios (95% confidence intervals) and P-values (reference group: low intake of lipids). Model, the model used for multivariable analysis; Without obesity, the model that included Charlson comorbidity index (cutoff ≥3), sex, alcoholic etiology, and recurrent AP; With obesity, the model that included these same variables plus obesity (body mass index ≥30 kg/m^2^); SFAs, saturated fatty acids; UFAs, unsaturated fatty acids; MUFAs, monounsaturated fatty acids; AP, acute pancreatitis*.

*Bold value indicates P-value statistically significant*.

## Discussion

Obesity is an important factor in AP severity and is associated with worse outcomes (3, 4, 19). The Atlantis prospective cohort study confirmed that obesity is associated with a higher mortality and with a higher incidence of POF (4), and fatty acids could be relevant mediators in the physiopathogenesis of moderate-to-severe AP in obese patients. There is robust evidence from basic research that UFAs are more toxic than SFAs (6, 7, 9, 12, 20–22). Analysis of postmortem tissue from obese patients has shown that UFAs are the predominant fatty acids in the pancreas, whereas the proportion of SFAs is lower (23, 24). Furthermore, UFAs are the most common (70–75%) non-esterified fatty acids in necrotic pancreatic collections (6–9). The Singh group has performed *in vitro* studies using pancreatic acinar cells and has studied animal models of AP and reports that UFAs are associated with increased inflammation and necrosis, while SFAs are not harmful (6, 7, 21, 22). These experiments suggest that (A) UFAs are the consequence of lipolysis of visceral fat by pancreatic lipases; (B) local UFAs produce necrosis of acinar pancreatic cells; and (C) uncontrolled UFA release results in high UFA levels in the bloodstream and is associated with renal tubular apoptosis (and subsequently with kidney failure) as well as with cell damage in lung alveoli that is similar to that seen in acute respiratory distress syndrome (6, 7, 9, 12, 22). UFAs seem to induce cell death by inhibiting mitochondrial complexes I and V and by increasing intracellular calcium release (6, 25). In addition, in severe AP a vicious cycle of inflammation and increasing oxidative stress is created. UFAs, but not SFAs, are sensitive to oxidative metabolic conditions and can worse this deleterious cycle in AP (26). This role of UFA-mediated lipotoxicity in worsening AP is consistent with the worse outcomes found in hypertriglyceridemic AP and in obese patients with AP (4, 27). On the other hand, studies show that dietary fatty acid patterns may affect body fat composition. Mice fed diets that have higher oleic acid and lower linoleic acid show differences in adipose tissue and fat composition of the pancreas (23). Studies in humans show that the proportion of different fatty acids of adipose tissue and plasma reflects, at least in part, different dietary intake patterns (28–30).

UFAs are more toxic than SFAs, and body fat composition is affected by dietary fatty acid intake. Thus, we hypothesized that regional fat intake patterns could be associated with different outcomes of AP. We also wanted to investigate whether dietary fat patterns had an independent effect on outcomes when we controlled for the presence of obesity. Accordingly, we compared outcomes in patients from regions of Spain with different fat intake levels. To our knowledge, this is the first study of its kind, there are no clinical studies studying this association in Spain or in other countries. Our study found that patients with AP from areas with a high UFA consumption had significantly higher rates of local complications, POF, mortality and frequency of moderate-to-severe AP in univariate analysis; this effect was also observed in multivariable analysis (for all four variables in the model not including obesity and for POF in the model including obesity). Patients from regions with high SFA intake did not have a worse prognosis. It is well-known that POF is the main determinant of severity in AP as reflected by the severe disease category of the revised Atlanta classification, which is defined exclusively by the presence of POF (15). Severe AP entails the greatest morbidity and mortality (2). Taken together, our results suggest that high UFA intake could be an important pre-morbid factor in determining the severity of AP (31).

Concerning the different types of UFAs, oleic acid is the more important MUFA, is the predominant fatty acid in human pancreatic extracts (23, 32) and in human pancreatic necrotic collections (6, 7). *In vitro* and laboratory animal studies suggest that oleic acid is associated with local and systemic toxicity (7, 10, 11, 33–35). In addition, in a study by Pironi et al. in patients with parenteral nutrition, the administration of a lipid emulsion based on MUFA produces a higher inflammatory status than in patients using a fish-oil based lipid emulsion (36). In the present study, residing in a region with high MUFA intake was independently associated with local complications and with moderate-to-severe AP in the model without obesity and with moderate-to-severe AP in the model that included obesity. In addition, two studies investigated halogenated fatty acids, a type of specific lipids produced from the oxidation of fatty acids by hypochlorous acid, in humans and in an animal model of AP. The animal model showed that the induction of AP produces an important release of free fatty acids from adipose tissue and also in the release of the chlorohydrins of oleic acid. Only the chlorohydrin of oleic acid was present in plasma (10). In humans, we found that oleic acid chlorohydrin was an excellent early marker of moderate-to-severe disease (11).

This study has several strengths, including the use of multicenter data and nationwide prospective data from patients with AP and the robust design and development of the ANIBES study. There are also some limitations: we used indirect data regarding dietary fat intake (regional consumption of fat according to the ANIBES study) instead of obtaining individual dietary data from every patient. It is difficult to conduct a prospective study that involves a similar sample of patients with detailed individual dietary information, and the lack of validated food questionnaires for the analysis of dietary fat and the different sources that are used in different countries make it difficult to perform an international study. Furthermore, dietary patterns are difficult to address in acutely ill patients, as this usually involves self-registry of food intake. Finally, a diet questionnaire is time-consuming, decreasing the feasibility of such a study due to the high number of patients required to reproduce our findings. However, we encourage the development of a multicenter prospective cohort study, which would provide more insights into the influence of lipids on AP. Besides, more nutritional data should be analyzed in further studies as PUFA, including the omega-6/omega-3 ratio that can be crucial in inflammatory processes. This is a limitation of the study although future studies by our group will include the omega-6/omega-3 ratio in order to have a more complete and global vision of the process. On the other hand, the *de novo* lipogenesis index (the synthesis of new fatty acids from non-lipid sources) will be evaluated in further studies by our research team. This index has a known correlation with metabolic disorders such obesity and type 2 diabetes and with inflammatory processes like moderate-to-severe AP. Finally, the influence of dietary fat patterns with possible similar pathophysiology could be studied in other severe diseases like a sepsis.

In conclusion, differences in regional dietary fat patterns seem to be associated with different outcomes of AP. Dietary fat patterns could represent a significant pre-morbid factor that determines the severity of AP. Moreover, UFAs, and particularly MUFAs, seem to be important mediators of the pathogenesis of the severity of AP.

## Data Availability Statement

The datasets generated for this study are available on request to the corresponding author.

## Ethics Statement

The studies involving human participants were reviewed and approved by Comité Ético de Investigación con Medicamentos del Hospital General Universitario de Alicante (PI2018-102). The patients/participants provided their written informed consent to participate in this study.

## Author Contributions

EM: conception, design, acquisition of data, analysis and interpretation of data, article drafting. GG-R: article drafting, acquisition of data, analysis and interpretation of data, and critical review for important intellectual content. GV-M: acquisition of data, analysis and interpretation of data, article drafting, and critical review for important intellectual content. ÁL and DC: article drafting and critical review for important intellectual content. ÁF, NB-L, JC, MB-B, AA-A, PL, MU-C, MS-C, JL-N, RG-F, CG-O, RI-U, CR-A, JS, MR-P, AM-Á, EO, MM, FB, JR-P, and SG-S: acquisition of data and critical review for important intellectual content. All authors gave final approval of the version to be published.

### Conflict of Interest

The authors declare that the research was conducted in the absence of any commercial or financial relationships that could be construed as a potential conflict of interest.

## Abbreviations

AP: acute pancreatitis
APFC: acute peripancreatic fluid collection
BMI: body mass index
CCI: Charlson comorbidity index
H: High consume
IQR: interquartile range
IRB: Institutional Review Board
L: Low consume
MUFA: monounsaturated fatty acid
OR: odds ratio
POF: persistent organ failure
PUFA: polyunsaturated fatty acid
SFA: saturated fatty acid
UFA: unsaturated fatty acid.
