# Supplementary material for: Dietary Fat Patterns and Outcomes in Acute Pancreatitis in Spain
Source: Front Med (Lausanne). 2020 Apr 9;7:126. doi: 10.3389/fmed.2020.00126 (PMC7160296; doi:10.3389/fmed.2020.00126)
Supplement: Supplementary file 1 [file Data_Sheet_1.docx]

Supplementary Table 1. Lipid caloric profile of the Spanish population stratified by region (data from the ANIBES study).

|  | Region | | | | | | | | |
| --- | --- | --- | --- | --- | --- | --- | --- | --- | --- |
|  | Barcelona | Canary Islands | Central | Levant | Madrid | North-East | North-West | North-Central | South |
| Lipids | 39.1 H | 36.7 L | 38.8 L | 38.2 L | 39.0 L | 39.4 H | 38.5 L | 40.3 H | 39.1 H |
| SFAs | 11.8 H | 11.7 L | 12.1 H | 11.2 L | 12.2 H | 11.9 H | 12.0 H | 12.0 H | 11.7 L |
| UFAs | 23.7 H | 21.2 L | 23.3 L | 23.6 L | 23.1 L | 23.8 H | 22.9 L | 24.2 H | 23.8 H |
| MUFAs | 17.1 H | 15.3 L | 16.7 L | 16.7 L | 16.7 L | 17.2 H | 16.2 L | 17.4 H | 17.2 H |
| PUFAs | 6.6 | 5.9 | 6.6 | 6.9 | 6.4 | 6.6 | 6.7 | 6.8 | 6.6 |

Data are shown as the regional mean percentages of total caloric intake. SFAs: saturated fatty acids. UFAs: unsaturated fatty acids. MUFAs: monounsaturated fatty acids. PUFAs: polyunsaturated fatty acids. H: High consume. L: Low consume. Based on data from 2009 Spanish citizens [17] that were retrieved from the ANIBES original database.

Supplementary Table 2. Number of centers and patients in the Atlantis database according to regions defined in the ANIBES study.

|  | Region | | | | | | | | |
| --- | --- | --- | --- | --- | --- | --- | --- | --- | --- |
|  | Barcelona | Canary Islands | Central | Levant | Madrid | North-East | North-West | North-Central | South |
| Centers, n (%) | 1 | 0 | 1 | 5 | 1 | 5 | 4 | 3 | 3 |
| Patients, n (%) | 59 | 0 | 51 | 439 | 77 | 352 | 237 | 272 | 168 |

The Atlantis database has data from 1,655 patients from 23 centers throughout Spain.
